# Applied *in situ* product recovery in ABE fermentation

**DOI:** 10.1002/btpr.2446

**Published:** 2017-03-10

**Authors:** Victoria Outram, Carl‐Axel Lalander, Jonathan G. M. Lee, E. Timothy Davies, Adam P. Harvey

**Affiliations:** ^1^ School of Chemical Engineering and Advanced Material, Newcastle UniversityNewcastle‐upon‐TyneU.K.; ^2^ Green Biologics Ltd45A Western Avenue, Milton ParkAbingdonOxfordshireU.K.

**Keywords:** In situ product recovery, ABE Fermentation, n‐Butanol

## Abstract

The production of biobutanol is hindered by the product's toxicity to the bacteria, which limits the productivity of the process. In situ product recovery of butanol can improve the productivity by removing the source of inhibition. This paper reviews in situ product recovery techniques applied to the acetone butanol ethanol fermentation in a stirred tank reactor. Methods of in situ recovery include gas stripping, vacuum fermentation, pervaporation, liquid–liquid extraction, perstraction, and adsorption, all of which have been investigated for the acetone, butanol, and ethanol fermentation. All techniques have shown an improvement in substrate utilization, yield, productivity or both. Different fermentation modes favored different techniques. For batch processing gas stripping and pervaporation were most favorable, but in fed‐batch fermentations gas stripping and adsorption were most promising. During continuous processing perstraction appeared to offer the best improvement. The use of hybrid techniques can increase the final product concentration beyond that of single‐stage techniques. Therefore, the selection of an in situ product recovery technique would require comparable information on the energy demand and economics of the process. © 2017 American Institute of Chemical Engineers Biotechnol. Prog., 33:563–579, 2017

## Introduction

Butanol is a commodity chemical used in a wide range of industries. It can be produced through biological or petrochemical routes. The original process route, started in 1913,^1^ was via the acetone, butanol, and ethanol (“ABE”) fermentation, but this lost favor to the petrochemical production process. Since the oil crisis in 1973 and the consequent dramatic rise in oil prices, alternative routes for petrochemical derivatives have been sought, sparking a renewal of interest in the ABE fermentation.^2^, ^3^

Fermentation originally ceased being the main production route for butanol when it became economically uncompetitive with the petrochemical production of butanol. The main reasons for this were high substrate cost, low solvent yield (approximately 2 wt%), and high energy requirement for butanol recovery by distillation.^2^, ^4^ The low yield and high energy requirement are closely related. One reason for the low yield is that the bacteria are inhibited by the butanol produced. This is a natural inherent limitation in the microorganism used for production. The low butanol tolerance necessitates low substrate concentrations, so as to maximize substrate consumption with minimal substrate loss. The low ABE concentration in the fermenter means that there is a high energy demand involved in the traditional distillation separation from a batch fermentation.^5^ This is compounded by the complex separation of butanol from water due to the azeotrope forming at 55.5 wt% butanol at 101.3 kPa.^6^

To overcome these problems, various strategies have been investigated, including: strain modifications to improve butanol tolerance,^2^, ^4^ *in vitro* production using immobilized enzymes,^7^ and fermentation process developments to remove the butanol from the fermentation broth as it is produced have been developed.^2^ Via such product removal techniques, the butanol concentration in the fermentation broth will be maintained below inhibitory levels, leading to increased productivity and overall titers. This would allow the process to be operated as a fed‐batch, or even continuous, fermentation.^8^ Relieving product toxicity will also have a significant impact on the economics of the fermentation. Increased productivity could allow a reduction in fermenter size, therefore a reduction in capital expenditure. *In situ* product recovery (ISPR) should increase the concentration of ABE for downstream processing, thereby reducing the energy demand and operational expenditure of the plant.

This paper provides a review of applied ISPR to ABE fermentations. The primary focus has been free cell (not immobilized or biofilm based) fermentations in a stirred tank reactor (STR), to allow for comparison of the various ISPR techniques. Other reactor configurations, such as immobilized bioreactors, have been considered if STR fermentations have not been performed. The techniques that have been experimentally combined with the ABE fermentation are gas stripping, vacuum fermentations, pervaporation, liquid–liquid extraction, perstraction, and adsorption.

## In Situ Product Recovery

The aim of ISPR techniques is to remove the product from the vicinity of the cell as soon as it is formed,^9^ this should lead to increased productivity and overall titers for inhibited fermentations and reduced waste water treatment costs.^10^ There have been several comprehensive reviews covering ISPR for a wide range of fermentations and products. Van Hecke *et al*.^11^ have provided the most recent review considering developments in ISPR between 2003 and 2013. They highlight that more research is required to prove scalability, long‐term robustness, and stability of the ISPR technology, decreased energy consumption and to maximize the product recovery^11^.

Since 2012, there has been a dramatic increase in the number of reviews focusing on the ISPR from the ABE fermentation. Abdehagh *et al*.^12^ focus on the separation ability of the technique, rather than improvements in production. This study concluded that pervaporation and adsorption show the most promise for ISPR. Huang *et al*.^13^ provide an overview of gas stripping, vacuum/flash separations, liquid–liquid extraction, membrane techniques, and adsorption, with a focus on novel separating agents such as ionic liquids and composite membranes. Xue *et al*.^14^ qualitatively compares ISPR techniques to conventional distillation, concluding that no ISPR technique will be able to concentrate the products to reagent grade. The most recent review was by Staggs and Nielsen,^8^ which focused on the mode of application of the ISPR technique, i.e., direct contact or recirculation in an external contactor. To complement these reviews, this review's focus is on the effect of each technique on the fermentation.

To compare the ISPR techniques, the amount of substrate utilized, productivity, and yield have been used. The substrate utilized is the total amount of substrate consumed during the fermentation. The productivity is defined here as the mass (g) of ABE produced per liter of reactor volume per hour. The yield is the mass (g) of ABE produced per mass (g) of substrate used.^15^ The % substrate utilized, productivity, or yield increase is the percentage difference between the substrate utilized, productivity or yield for the integrated *in situ* recovery fermentation and the non‐integrated (control) fermentation. These parameters have been selected as they are generally considered as the main parameters of comparison in experimental based literature, particularly productivity and yield. Substrate utilization was selected to demonstrate the improvements in the fermentation, particularly fermentation longevity due to reduced toxicity. Measurement of substrate can be considered more reliable compared to product concentration, which can be highly inaccurate based on product separation methods. The concentrated product is not always directly measured, sometimes being inferred from model solution data or assuming the yield is the same as the control fermentation 16. Also, the final concentration varies based on volume used for calculation, i.e., fermentation volume (which is variable during the fermentation) through to the condensate concentration post separation, particularly with evaporative techniques. Productivity and yield provide a standardized measure of the fermentation performance. By comparing the % increase compared to the control fermentation the effects of various differences in experimental methods should be negated, allowing trends relating to the impact of the ISPR technique on the fermentation to be observed.

This review differs from the previous reviews by taking a quantitative approach to the comparing the experimental data of the ISPR techniques and their impact on the fermentation. This review also considers the final concentration from each ISPR technique that will enter the downstream distillation process, where possible.

### Gas stripping

Gas stripping is a separation technique that involves the removal of solvents via dissolution into a gas passing through the fermentation broth. This technique was studied by a range of authors from the mid 1980s (e.g., Ennis *et al*.^17^), through to the present day (e.g., Xue *et al*.^18^). Numerous publications cover all operation modes and a range of bioreactor configurations.^19^, ^20^, ^21^, ^22^

Gas stripping for ABE fermentations involves the recycling of the fermentation gases (carbon dioxide and hydrogen), or application of other anaerobic gases such as oxygen‐free nitrogen,^17^, ^23^, ^24^ through the fermenter via a condenser to remove the ABE from the gas stream.^25^ As it can be performed *in situ* without the need for expensive equipment and plant modifications, gas stripping is considered a simple technique.^26^ Based on data from Ezeji *et al*.,^22^ the concentration in the gas stream is very dilute at approximately 1.7 mg/L, meaning that large condensing duties will be required, which will significantly increase operating costs. Additionally, the compressor duty to supply gas at flow rates of 2–3 vvm of a plant‐scale reactor is energy intensive.^17^

A wide range of studies have been performed for gas stripping, with Ezeji's body of work^5^, ^22^, ^27^, ^28^, ^29^ being the most comprehensive. A general conclusion to be drawn from this data is that the productivity of the fermentation is improved through the application of gas stripping. The productivities in Table 1 show an increase moving from batch to fed‐batch. It must be noted that the productivity increase seen by Maddox *et al*.,^30^ 357%, is due to the low productivity of the control fermentation (0.07 g ABE/Lh). This demonstrates that relieving product inhibition has a significant positive effect on the fermentation. This removal of product toxicity has allowed for more substrate to be consumed, with more than a 100% increase in substrate utilization possible.

**Table 1:** Free Cell ABE Fermentation Performance with *in situ* Product Recovery by Gas Stripping in an STR

Mode	Microorganism	Substrate (Concentration for ISPR)	% Substrate Increase for ISPR (vs. control)	ABE Productivity for ISPR (g ABE/L.h)	% Productivity Increase (vs. Control)	Yield for ISPR (g ABE/g Substrate)	% Yield Increase (vs Control)	Gas^a^	Ref.
Batch	*Clostridium acetobutylicum* P262^b^	Lactose (58 g/L)	101%	0.31	41%	0.27	−31%	N_2_	17
	*C. acetobutylicum* P262 b	Whey Permeate/Lactose (199 g/L)	542%	0.32	357%	0.35	35%	CO_2_+H_2_	30
	*Clostridium beijerinckii* BA101	Glucose (162 g/L)	263%	0.6	107%	0.47	21%	CO_2_+H_2_	22
	*C. beijerinckii* BA101	Liquefied Corn Starch (LCS) (55 g/L)	23%	0.31	107%	0.43	5%	CO_2_+H_2_	29
	*C. beijerinckii* BA101	Saccharified Liquefied Corn Starch (SLCS) (64 g/L)	41%	0.4	74%	0.41	2%	CO_2_+H_2_	29
	*C. beijerinckii* CC101	Wood Pulp Hydrolysate (33 g/L)	36%	0.17	55%	0.39	18%	CO_2_+H_2_	31
	*C. beijerinckii* NRRL B593 c	Glucose/Xylose (60 g/L)	77%	0.29	81%	0.32 d	‐	CO_2_+H_2_	16
Fed‐ Batch	*C. beijerinckii* BA101	Glucose (500 g/L)	1001%	1.16	300%	0.47	21%	CO_2_+H_2_	5
	*C. beijerinckii* BA101	Saccharified Liquefied Corn Starch (SLCS) (226 g/L)	395%	0.59	157%	0.36	−10%	CO_2_+H_2_	29
	*C. acetobutylicum* P262 b	Whey Permeate (183 g/L)	576%	0.26	271%	0.38	19%	CO_2_+H_2_	25
Continuous	*C. beijerinckii* BA101	Glucose (1125 g/L) e	2278%	0.92	229%	0.41	5%	CO_2_+H_2_	27
	*C. beijerinckii* NRRL B593 c	Glucose/Xylose (52 g/L) e	56%	0.93	40%	0.30 c	‐	N_2_	16
	*C. beijerinckii* NRRL B593 c	Glucose/Xylose (41 g/L) e	88%	1.3	65%	0.30 c	‐	N_2_	16

a CO_2_ and H_2_ represent recycling of the gases produced during fermentation.

b *C. acetobutylicum* P262 has since been reclassified as *Clostridium saccharobutylicum* P262.^32^

c *C. beijerinckii NRRL B593* produces isopropanol instead of acetone.^16^

d Yield has been assumed equal to the yield of the batch for calculations of the productivity.^16^

e The dilution rate was 0.003 h^−1^, 0.06h^−1^, and 0.11 h^−1^, respectively.

Interestingly, there is a decrease in productivity when moving to a continuous fermentation compared to the fed‐batch fermentation, but it should be noted that this comparison is based upon only one strain *C. beijerinckii* BA101. In the continuous fermentations performed by Ezeji *et al*.,^27^ this decrease in productivity cannot be related to the decrease in yield (0.92 g/L.h and 0.41 g/g for continuous^27^ compared to 1.16 g/L.h and 0.47 for fed‐batch^5^), because if the yield was the same as in the fed‐batch fermentation the productivity would still be lower. Low productivity is a result of the cyclic fermentation profile, switching between the acidogenic and solventogenic phase, which means that a true steady state is not attained. The reduced yield is due to the removal of some of the nutrients from the process, in the reactor bleed, meaning 100% sugar utilization was not possible.^27^

There are some results in Table 1 which show the yield of the gas stripping process being greater than the theoretical yield of the bacteria. In the work by Ezeji *et al*.,^5^, ^22^, ^27^ the increased yield, 0.41–0.47, is contributed to the consumption of other carbon sources present in the complex medium used, such as sodium acetate. It is also suspected that less substrate is used for biomass maintenance, therefore a greater product yield is possible.^5^ No reason was provided for the higher than expected yield in the case of Ezeji *et al*.^29^ In some cases, a decrease in yield compared to the non‐integrated fermentation is seen, for example Ennis *et al*.^17^ observed a 31% decrease in yield; but this is probably related to inefficient condensing capability, meaning that not all solvents are captured and are consequently not accounted for when calculating the yield, as seen by Groot *et al*.^24^ and Ezeji *et al*.^5^ who take into account solvent losses when calculating the overall yield. This inability to capture all the solvents has a knock‐on effect, meaning that the productivities cannot be assumed to be accurate, adding further uncertainty to any comparison between operating modes. de Vrije *et al*.^16^ overcame the loss of products by assuming the same yield as the control fermentation, 0.30–0.32 g/g, and used this to calculate the productivity. This calculation method is likely to provide an inaccurate result as it is assumed that the ISPR technique has no negative or positive effect on the microorganism's performance.

Gas stripping has limitations due to the low ABE concentration in fermentation broth, large quantities of water removed and high gas flow rates required.^33^ Xue *et al*.^33^ proposed that operating at higher butanol concentrations, 8 g butanol/L compared to 5 g butanol/L, would increase the concentration of product in the vapor and reduce the energy for separation. The downside to this is 8 g butanol/L is often inhibitory to the bacteria. Xue *et al*. 33 tested this idea in an immobilized fermentation using an intermittent gas flow rate. The gas flow only operated while the butanol concentration in the broth was greater than 8 g butanol/L. This gas stripping regime saw a 33% increase in productivity compared to the control, while the yield remained constant. Stripping at a higher concentration saw a condensate concentration of 195.9 g ABE/L, compared to 76.8 g ABE/L achieved by Ezeji *et al*.^29^ in a fed‐batch free cell fermentation with saccharified liquefied cornstarch with the butanol maintained no higher than 5 g butanol/L.

#### Hybrid Gas Stripping

Since 2013 there has been a flurry of investigations into hybrid separations. A major focus of the hybrid separation processes has been improving the efficiency of gas stripping. This has included investigations into multi‐stage gas stripping processes, increasing the temperature at which gas stripping is performed and hybrid gas‐stripping pervaporation processes,^16^, ^18^, ^19^, ^34^, ^35^, ^36^ with the aim of reducing the energy requirements for further separation of the condensate. Oudshoorn *et al*.^37^ estimated the selectivity of gas stripping for butanol to be 4–22, which is low compared to distillation with an estimated selectivity of 72, thereby the recovered solution is not very concentrated. It has been widely noted that to achieve significant decreases in the energy for downstream purification, two‐phase separation needs to be observed in the recovered ABE solution.^19^, ^21^, ^31^ To achieve this phase separation, it has been suggested that the butanol concentration in the fermenter should be greater than 8 g/L, but concentrations this high start to impact on the fermentation performance.^19^, ^34^

To achieve this higher concentration, Xue *et al*.^36^ proposed a two‐stage gas stripping process. The aqueous phase condensate from the first stripping stage, 153 g ABE/L, being subjected to gas stripping to achieve a more concentrated solution, 447 g ABE/L. When combined with the organic phase from the first stripping stage the final product solution was 532 g ABE/L.^36^ The first stage reduced inhibition in the fermenter, while the second stage increased the concentration of condensate. Xue *et al*.^34^ proceeded to further optimize the process and achieved a final product concentration of 671 g ABE/L, predicting a 50% decrease in operational energy to 7–15 MJ/kg butanol.^14^de Vrije *et al*.^16^ discussed the use of increasing the temperature while gas stripping to improve the selectivity of the process. de Vrije *et al*. 16 utilized the bacteria's natural sporulation cycle for a repeated batch process. The broth was heated to 70°C at the end of a batch to remove the products via enhanced gas stripping and heat shock the spores to restart the fermentation with fresh media added. The final condensate concentration, nor total product formation was not stated so this cannot be compared to the two‐stage process proposed by Xue *et al*.^34^ Chen *et al*.^19^ also investigated the use of a higher stripping temperature, but combined the fermentation with an immobilized cell bioreactor. Immobilization of the cells allowed the fermentation medium to be heated to 70°C without impacting the viability of the bacteria. This saw condensate concentrations of 703 g butanol/L in the organic phase and 78 g butanol/L in the aqueous phase. The combined concentration was 150 g butanol/L 19, indicating that a two‐stage stripping system will offer better performance.

Gas stripping has also been combined with pervaporation, using a carbon nanotube filled polydimethylsiloxane (CNT‐PDMS) membrane.^18^ Gas stripping was first performed on the fermentation broth to relieve ABE toxicity. Pervaporation was then performed on the aqueous phase portion of the condensate to further increase the final product concentration. This method produced a final product concentration of 623 g ABE/L,^18^ which is slightly lower than that achieved using the two‐stage gas stripping process (671 g ABE/L).^34^ Xue *et al*.^18^ predict that the energy for the pervaporation step will be as low as 4 kJ/kg butanol due to the starting solution containing 80 g/L butanol. The overall two‐stage gas stripping‐pervaporation process would require ∼20 MJ/kg butanol. Compared to two‐stage gas stripping, a hybrid gas stripping‐pervaporation process is more complex, producing a lower product concentration and requires more energy for this stage of the process.

These hybrid techniques apply a second/enhanced separation stage to the fermentation. Other than de Vrije *et al*.,^16^ have all focused on immobilized fermentations. It would be useful to see the potential impact these hybrid techniques (if possible) could have when combined with free cell fermentations. Liu *et al*.^38^ and van der Merwe *et al*.^39^ proposed flowsheets with alternative product concentration techniques to distillation for the ABE fermentation. It would be advantageous to complete a similar analysis for the various hybrid options to help decide which further concentration techniques would be best suited for an industrial process.

### Vacuum fermentation

Vacuum fermentations have a reduced pressure in the fermenter, causing the ABE to “boil off” at fermentation temperature. Vacuum fermentations were first used in the ethanol industry to selectively remove ethanol from fermentation broths. The use of a vacuum for an ABE fermentation should be more straightforward than for an ethanol fermentation, as the *Clostridium sp*. used are strict anaerobes.^40^ The viability of vacuum fermentations was experimentally tested by Mariano *et al*.^40^, ^41^, ^42^

Mariano *et al* 40 demonstrated that it is possible to recover ABE from fermentation broths under vacuum on a laboratory scale with no adverse effects on the bacteria. The system was initially characterized using a model ABE solution, with concentrations ranging from 5–15 g butanol/L, but this was found to be unrepresentative of real fermentation broths in which the gas created by the bacteria expands under reduced pressure, stripping the solvents from the broth. This effectively creates a hybrid gas stripping‐vacuum system. Mariano *et al*.^41^ reported that under constant vacuum conditions, the rate of removal of butanol was approximately 10 times higher than that found by Ezeji *et al*.^22^ using gas stripping, which could reduce the butanol concentration by up to 68.5%.^41^ Performing the fermentation under vacuum was able to achieve butanol concentrations of less than 1 g/L in the fermentation broth.^40^ More recently, vacuum fermentation was also proven to be effective with combining with simultaneous saccharification, fermentation, and recovery.^43^ Qureshi *et al*.^43^ successfully demonstrated the combined process using 86 g/L corn stover as a feedstock, in simultaneous saccharification, fermentation, and recovery. The ability to utilize lignocellulosic feedstocks as well as combining feedstock treatment with the fermentation and recovery should also see a reduction in capital and operational costs.

Two vacuum modes have been investigated: constant and cyclic. Cyclic vacuum fermentations were found to be considerably more competitive in terms of energy demand than traditional distillation. The cyclic vacuum process allows the concentration of butanol to build up, then reduces the concentration rapidly by applying a vacuum for 2 h, repeating this process throughout the fermentation.^42^ This is the only variation in operation that has been investigated. Currently, all trials have been on batch fermentations (Table 2) with a maximum applied vacuum time of 30 h,^40^ so whether vacuum fermentation can be extended for improved productivity is unknown. Whether this extended time at reduced pressure would impact microbial performance is also unknown.^40^, ^41^, ^42^

**Table 2:** Free Cell ABE Fermentation Performance with *in situ* Product Recovery by Vaccum in an STR

Mode	Microorganism	Substrate (Concentration for ISPR)	% Substrate Increase for ISPR (vs. control)	ABE Productivity for ISPR (g ABE/L.h)	% Productivity Increase (vs. control)	Yield (g ABE/g Substrate)	% Yield Increase (vs. control)	Operating Temperature (°C)	Vacuum Range (mmHg)	Vacuum Operating Mode	Ref.
Batch	*C. beijerinckii* P260	Glucose (62 g/L)	38%	0.34	31%	0.24	−31%	37	711–737	Intermittent	40
	*C. beijerinckii* NCIMB 8052	Glucose (66 g/L)	56%	0.37	54%	0.34	−8%	35	711–737	Intermittent	42
	*C. beijerinckii* P260	Glucose (58 g/L)	29%	0.28	8%	0.22	−37%	37	711–737	Continuous	10
	*C. beijerinckii* NCIMB 8052	Glucose (65 g/L)	54%	0.43	79%	0.29	−22%	35	711–737	Continuous	41
	*C. beijerinckii* P260	Corn Stover (83 g/L)	7%	0.34	55%	0.39	30%	35	584	Continuous	43

All of the literature focusing on vacuum fermentations for the ABE process is a product of the same researchers.^40^, ^41^, ^42^, ^43^ In each of these studies, inefficient condensation resulted in low ABE capture with ABE condensate concentration of 16–49 g/L, lower than the concentration required for spontaneous phase separation.^40^ Consequently, the yield has been underestimated as demonstrated by the negative yield increases seen in Table 2. Qureshi *et al*.^43^ accounted for losses of ABE in the system, based on previous work, and therefore achieved a positive yield increase of 30%. This appears to be a common flaw in evaporative techniques, in particular vacuum fermentation and gas stripping.^5^, ^43^ Another potential problem with the use of vacuum fermentations, highlighted by Mariano *et al*.,^40^, ^42^ was that a small concentration of acids (up to 0.4 g/L) was detected in the condensate. As the fermentation utilizes acids as intermediates, acid removal is undesirable during ISPR as it will reduce the yield of the process.

Mariano *et al*.^42^ assessed the energy requirement for the addition of a vacuum to the fermentation. They showed that the use of a vacuum reduced the downstream distillation energy requirement by 11.2 MJ/kg butanol for a continuous vacuum and 15 MJ/kg butanol for intermittent vacuum. When combined with the energy required for the vacuum fermentation, the total energy requirement became 32.4 and 22.0 MJ/kg butanol for continuous and intermittent vacuum, respectively. For a comparable batch process without ISPR, the energy requirement was 26.8 MJ/kg butanol. The use of a continuous vacuum will see an increase in the plant energy demand, defeating one of the main purposes of adding ISPR. The use of an intermittent vacuum sees an 18% decrease in energy. Mariano *et al*.^44^ have demonstrated that as the butanol concentration in the first distillation column increases, the energy requirements rapidly decrease. This can be inferred as the reason the intermittent vacuum fermentation requiring less energy than the continuous vacuum fermentation, where the condensate concentrations were 51.5 g ABE/L and 33 g ABE/L, respectively 42.

Generally, the conclusions over application of vacuum to ABE fermentations are not definitive, as there is not enough data (Table 2). The application of a vacuum can increase the substrate utilization and productivity of the fermentation, but without an efficient product capture step the benefits are not observed, as significant product is lost. The decreased yield would have a negative effect on the process economics, impacting the amount of feedstock required.

### Pervaporation

**Figure 1:**
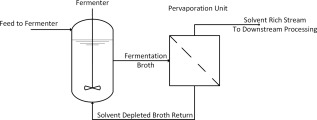
Diagram of a fermentation with *in situ* product recovery by pervaporation.

Pervaporation utilizes a membrane between the fermentation broth and the gaseous phase,^45^ a simplified schematic is shown in Figure 1. Pervaporation renders the flowsheet more complex, as generally, an external unit is required to perform the separation. It is possible for pervaporation to be performed within the bioreactor,^46^, ^47^ but this is an unusual configuration.

Pervaporation has been shown to increase the substrate utilization, productivity, and yield of ABE fermentations (see Table 3). It is the most widely researched area in relation to ISPR and the ABE fermentation. Within this body of research, there is a greater focus on membrane performance than integrated fermentation performance.^48^ Of the work performed where the ABE fermentation is coupled to a pervaporation system, no definitive conclusion can be drawn on fermentation or pervaporation operating conditions.

**Table 3:** Free Cell ABE Fermentation Performance with *in situ* Product Recovery by Pervaporation in an STR

Mode	Microorganism	Substrate (Concentration for ISPR)	% Substrate Increase for ISPR (vs. Control)	Productivity for ISPR (g ABE/Lh)	% Productivity Increase (vs. Control)	Yield for ISPR (g ABE/g Substrate)	% Yield Increase (vs. Control)	Membrane	Driving Force	Driving Force Rate	Ref.
Batch	*C. beijerinckii* BA101	Glucose (60 g/L)	5%	0.5	47%	0.42	42%	Silicone tubing	Air	2–18 L/min	51
	*C. acetobutylicum* XY16	Glucose (60 g/L)	15%	0.3	50%	0.37	29%	PDMS/ceramic composite	Vacuum	<400Pa	59
	*C. saccharo‐butylicum* P262	Whey Permeate/Lactose (211 g/L)	424%	0.43	207%	0.37	19%	Silicone	N_2_	40–42 L/h	67
	*C. acetobutylicum* DP217	Cassava (70 g/L)	0%	0.51	21%	0.36	9%	Silicalite‐PDMS/PAN	Vacuum	280 Pa	58
Fed‐Batch	*C. acetobutylicum* ATCC824	Glucose (199 g/L)	138%	0.66	47%	0.27	−18%	PDMS	Vacuum	200 Pa	52
	*C. acetobutylicum* ATCC824	Glucose (276 g/L)	231%	0.94	109%	0.28	−15%	SDS	Vacuum	200 Pa	52
	*C. acetobutylicum* XY16	Glucose (200 g/L)	285%	0.21	5%	0.28	−3%	PDMS/ceramic composite	Vacuum	<400Pa	59
	*C. acetobutylicum* XY16	Glucose (200 g/L)	285%	0.25	25%	0.3	3%	PDMS/ceramic composite	Vacuum	<400Pa	59
	*C. beijerinckii* BA101	Glucose (384 g/L)	568%	0.98	180%	0.43	−2%	Silicone	Air	2–18 L/min	68
	*C. acetobutylicum* ATCC824	Glucose (445 g/L)	950%	0.18	33%	0.35	21%	Silicalite‐Silicone	Vacuum	266‐666 Pa	57
	*C. acetobutylicum* DP217	Cassava (736 g/L)	951%	0.76	80%	0.38	15%	Silicalite‐PDMS/PAN	Vacuum	280 Pa	58
	*C. acetobutylicum* P262 a	Whey Permeate (123 g/L)	355%	0.14	100%	0.34	6%	Poly‐propylene	N_2_	10–20 L/min	25
	*C. acetobutylicum* ATCC 55025	Glucose (172 g/L)	144%	0.46	15%	0.32	3%	Zeolite‐PDMS	Vacuum	<1 kPa	60
Cont‐inuous	*C. acetobutylicum* ATCC824	Glucose (100g/L)/Xylose (50 g/L) b	201%	0.65	126%	0.30	67%	PDMS (Pervatech)	Vacuum	960 Pa	53

a *C. acetobutylicum* P262 has since been reclassified as *C. saccharobutylicum* P262.^32^

b The overall dilution rate was 0.0017 h^−1^.

For pervaporation, the major decision to be made is the choice of membrane, as the ideal membrane should selectively allow the transfer of ABE while retaining butyric acid, acetic acid, water, and nutrients. The membrane also needs to be minimally fouling, so that it is not blocked by cells adhering to the membrane surface. Table 3 shows that a range of organophilic membranes have been tested, all of which show an improvement in the productivity of the fermentation. While silicone (including polydimethylsiloxane, PDMS) has been the most investigated membrane, as it is commercially available, inexpensive, and offers easy manipulation for the development of laboratory‐scale pervaporation units,^45^, ^46^, ^49^, ^50^, ^51^, ^52^, ^53^, ^54^ other membrane choices, including polypropylene,^25^ oleyl alcohol liquid membrane on a polypropylene support,^55^ polystyrene‐*b*‐polydimethylsiloxane‐*b*‐polystyrene (SDS),^52^ and PDMS‐supported ionic liquid membranes^56^ have been investigated. More recently, there has been a move toward the use of composite membranes, utilizing a combination of materials for improved selectivity and flux performance. This has included silicalite‐silicone composite membrane^57^ silicalite‐PDMS/polyacrylonitrile (PAN) membrane,^58^ PDMS/ceramic composite,^59^ zeolite‐mixed PDMS^60^ and carbon nanotube filled PDMS (CNT‐PDMS).^61^ A wider range of membranes have been investigated for butanol/water or ABE/water solutions, but this does not necessarily transfer to the performance in conjunction with a fermentation.^48^

The membrane choice affects the selectivity and diffusion rates of the ABE, which will determine the concentration of the ABE in the permeate. Unfortunately, the selectivity is in competition with the flux. Improvements in flux can be made to pervaporation through the use of higher temperatures, for example, but this would require an additional step of microorganism removal prior to heating the pervaporation feed stream (to 65–78°C), and cooling of the retentate prior to re‐addition to the bioreactor.^62^, ^63^ Other factors such as feed concentration and composition, biomass concentration, and sweep gas flow rate also influence the membrane flux.^51^, ^64^ Qureshi and Blaschek^51^ and Gapes *et al*.^65^ state that the application of a vacuum on the permeate side increased the flux compared to the application of a sweep gas, which explains why recent research has focused on vacuums.^59^, ^62^, ^66^

The use of a composite membrane has also been proposed as a method of achieving non‐competitive flux and selectivity. Polymers have high flux, are relatively cheap and easy to fabricate into a membrane, but are prone to aging over a long time. Inorganic materials are often highly selective for butanol and have good strength but are expensive. By combining both materials together, the membrane should be more selective with a sufficient flux while not having a prohibitive cost for scale‐up.^13^ Li *et al*.^58^ used a silicalite‐PDMS/PAN membrane and were able to achieve an average permeate concentration of 201 g ABE/L, resulting in spontaneous phase separation. Xue *et al*.^61^ used a CNT‐PDMS membrane. They were able to achieve a butanol separation factor of 16.6 and butanol titers over 100 g butanol/L in the permeate, when the membrane consists of 10% carbon nanotubes. This membrane has not been directly applied to the ABE fermentation. Zeolite‐PDMS were also tested by Xue *et al*.,^60^ with an 80% zeolite loaded PDMS membrane being combined with a free cell fermentation. The condensate collected contained 253 g ABE/L, which formed an organic phase containing over 600 g butanol/L. The membrane surface was smooth and non‐porous, this reduced fouling by the bacteria as there were no pores or imperfections for the bacteria to stick to.^60^ The addition of a second material to increase the selectivity and flux significantly improves the compatibility with the ABE process. Combine this with achieving high purity ABE (greater than 250 g ABE/L in the total permeate) means the downstream energy required for product recovery would decrease.^58^ Currently, these membranes have only been made for research purposes. An economic study is required to understand the impact the use of these novel membranes would have on the process.

As pervaporation is an evaporative ISPR technique, the product is captured through condensation. The inefficiencies of condensation of ABE have already been discussed in the sections concerning gas stripping and vacuum fermentation. In the papers regarding these techniques, the inefficiencies of complete product capture are acknowledged, but this has not been the case for pervaporation. In Table 3, it can be observed, for fed‐batch fermentations, a negative yield increase compared to the control fermentation. The fed‐batch fermentations are not inhibited by ABE or limited by available substrate, as an increase in substrate utilization is observed, yet there is a decrease in yield compared to the control fermentation. This indicates the potential loss of product, possibly through incomplete condensation. Incomplete product capture appears to be an inherent issue where the ABE is transferred to the vapor phase and needs to be considered when designing the fermentation process.

### Liquid‐liquid extraction

Liquid‐liquid extraction (LLE) is a common technique in the processing industries. It exploits the differences between relative solubility of a compound in two immiscible components. Typically, the solvent used is an organic liquid which is immiscible in water, therefore when applied to a fermentation broth, the product will preferentially transfer from the aqueous phase into the organic phase.

There are a variety of key parameters that need to be considered, described in Table 4. Davison and Thompson^69^ also stated that the operability of the LLE system and the method of contacting must be considered, especially for plant or pilot plant operation, which was only considered by Roffler *et al*.^70^ One of the biggest challenges with LLE is finding an extractant which can satisfactorily meet all of the key characteristics for the extractant.

**Table 4:** Key Characteristics for LLE Extractant

Key Characteristic	Ref.
Non‐toxic to the microorganism	69, 71
Have a high partition coefficient (high capacity)	69, 71
Immiscible with water and not emulsion‐forming with aqueous phase	71
Favorable physical properties, for example a low viscosity and a large density difference compared to water	71, 72
High chemical stability, particularly at high temperatures (to ease extractant renewal)	72
Sterilizable	71
Commercially available at low cost	71, 72, 73

Ishii *et al*.^71^ and Roffler *et al*.^74^ performed a series of extractive batch fermentations to find a suitable extractant for the ABE fermentation, and both concluded that oleyl alcohol is an acceptable extractant for butanol. It is non‐toxic to the microorganisms and has a good distribution coefficient with an average distribution coefficient for butanol of 4.4.^74^ Table 5 shows that oleyl alcohol increases both yield and productivity compared to a non‐integrated fermentation, unlike other tested extractants. As a consequence of this, oleyl alcohol is the most widely studied extractant, with Roffler *et al*.^74^ performing batch fermentations and fed‐batch fermentations,^75^ before moving to a scale down industrial fed‐batch system^70^ (the outcomes of these are shown in Table 5) and an economic assessment of a commercial process of a continuous ABE fermentation with an inline recovery unit using oleyl alcohol.^76^ The fed‐batch fermentations demonstrate an improvement over integrated batch fermentations, with substrate utilization and productivity increases over 100% being achieved.^75^ Roffler *et al*.^74^, ^75^ reported final organic phase butanol concentrations of 24–30 g butanol/L. This is a small concentration increase, especially compared to those seen in the condensate for the evaporative techniques. ABE separation from the oleyl alcohol should be less intensive than separation from water, as there is no water‐butanol/water‐ethanol azeotrope formations, but the low concentration will impact the energy requirement.

**Table 5:** Free Cell ABE Fermentation Performance with *in situ* Product Recovery by Liquid‐Liqud Extraction in an STR

Mode	Microorganism	Substrate (Concentration for ISPR)	% Substrate Increase for ISPR (vs. Control)	Productivity for ISPR (g ABE/Lh)	% Productivity Increase (vs. Control)	Yield for ISPR (g ABE/g Substrate)	% Yield Increase (vs. Control)	Extractant	Ref.
Batch	*C. acetobutylicum* ATCC824	Glucose (97 g/L)	18%	0.69	19%	0.17	−6%	Kerosene	74
	*C. acetobutylicum* ATCC824	Glucose (78 g/L)	−5%	0.53	−9%	0.17	−6%	50wt% Dodecanol in kerosene	74
	*C. acetobutylicum* ATCC824	Glucose (89 g/L)	9%	0.43	−26%	0.16	−11%	30wt% Tetradecanol in kerosene	74
	*C. acetobutylicum* ATCC824	Glucose (100 g/L)	22%	0.72	24%	0.19	6%	Oleyl Alcohol	74
	*C. acetobutylicum* ATCC824	Glucose (100g/L)	22%	0.71	22%	0.17	−6%	50wt% Oleyl alcohol in decane fraction	74
	*C. acetobutylicum* ATCC824	Glucose (100 g/L)	22%	0.74	28%	0.18	0%	50wt% Oleyl alcohol in benzyl benzoate	74
	*Clostridium saccharoper‐butylacetonicum* N1‐4	Potato glucose (75 g/L)	34%	0.52	2%	0.38	0%	Oleyl Alcohol	87
	*C. saccharoper‐butylacetonicum* N1‐4	Potato glucose (74 g/L)	32%	0.55	8%	0.4	5%	Methylated crude palm oil (CPOE)	87
	*C. acetobutylicum* BCRC10639 (ATCC824)	Glucose (unknown)		0.27	28%	0.21	15%	Biodiesel	88
	*C. acetobutylicum* ATCC824	Glucose (86 g/L)	51%	0.36	9%	0.36	−8%	Oleyl Alcohol	86
	*C. acetobutylicum* ATCC824	Glucose (117 g/L)	105%	0.46	39%	0.37	−4%	Oleyl Alcohol with gas stripping	86
Fed‐Batch	*C. acetobutylicum* ATCC824	Glucose (155 g/L)	91%	0.9	55%	0.24	33%	Oleyl Alcohol	75
	*C. acetobutylicum* ATCC824	Glucose (218 g/L)	169%	1.5	159%	0.22	22%	Oleyl Alcohol	75
	*C. acetobutylicum* ATCC824	Glucose (303 g/L)	274%	1.3	124%	0.21	17%	Oleyl Alcohol	75
	*C. acetobutylicum* ATCC824	Glucose (86 g/L)	60%	0.17	−23%	0.23	−21%	PPG1200	83
	*C. acetobutylicum* BCRC10639 (ATCC824)	Glucose (unknown)		0.30	37%	0.31	65%	Biodiesel	88
	*C. acetobutylicum* P262 a	Whey Permeate (68.6 g/L)	154%	0.15	114%	0.35	9%	Oleyl Alcohol	25
	*C. acetobutylicum* ATCC824	Glucose (300 g/L)	270%	1	72%	0.19	6%	Oleyl Alcohol	70

a *C. acetobutylicum* P262 has since been reclassified as *C. saccharobutylicum* P262.^32^

Other solvents have been tested with fermentations, such as decanol, dibutylphthalate, 2‐butyl‐1‐octanol, and poly (propylene glycol) 1200.^77^, ^78^, ^79^, ^80^, ^81^, ^82^, ^83^ Decanol has a high distribution coefficient for butanol, 6.2, but is toxic to the bacteria and dissolves into the fermentation broth.^78^, ^79^ Evans and Wang^79^ investigated a mixture of decanol‐oleyl alcohol as an extractant, where it was observed that a mixture containing 40% of decanol was detrimental to fermentation. On the other hand, Bankar *et al*.^81^ did perform a successful continuous fermentation with a 20% decanol, 80% oleyl alcohol mixed extractant with a two‐stage immobilized reactor. A maximum solvent productivity of 2.07 g/L.h was achieved in the second stage reactor at a dilution rate of 0.5 h^−1^.The downside of this was the final product concentration only reached 25.32 g ABE/L. Dibutylphthalate was used as an extractant by Wayman and Parekh^77^ but Roffler *et al*.^74^ ruled out its use in a fermentation due to the density being very similar to water making the removal of the extractant from the fermentation broth difficult. Barton and Daugulis^83^ screened 63 organic solvents and decided that poly (propylene glycol) 1200 (PPG) was the best extractant. This was largely due to the high partition coefficient and biocompatibility of the extractant. Unfortunately, PPG did not show the same promise in fed‐batch fermentations as a reduction in both productivity (−23%) and yield (−21%) was seen, Table 5. The authors have associated this with the extraction of acids and intermediates into the PPG as the glucose uptake rate had increased by 60% compared to the control, with only a 26% increase in solvent formation.^83^ Extraction of acids is not a desirable trait in the extractant, as the acids cannot be assimilated into the desired products. 2‐butyl‐1‐octanol was suggested as an extractant as a result of González‐Peñas *et al*.^84^ extractant screening method. It was selected as it was shown to be biocompatible, with an increase in yield over the control experiment. Surprisingly, González‐Peñas *et al*.^84^ found 2‐butyl‐1‐octanol to be a better extractant than oleyl alcohol which has been the go‐to extractant for ISPR. 2‐butyl‐1‐octanol produced a yield of 27.43 w/w% compared to 25.5 w/w% for oleyl alcohol. It also had a greater distribution coefficient (6.76) and selectivity (644) for butanol compared to oleyl alcohol (4.57, 295) 84.

With LLE, the achieved yields are very low (Table 5), especially compared with evaporative techniques like gas stripping and pervaporation (Tables 2 and 3). With evaporative techniques, the average yield is 0.35 g ABE/g substrate which is close to the theoretical yield. The average yield for LLE is 0.25 g ABE/g substrate, which is approximately 40% lower than evaporative techniques. This reduction in yield between techniques is likely to be related to the contact of extractant with fermentation broth. Either substrate or acids is being removed from the broth into the extractant or the extractant is having a toxic effect. While these extractants have been selected due to being biocompatible, the sustained contact over the duration of the fermentation could be having a negative impact. In contrast, there is a general improvement in productivity over the control fermentation; therefore, further work would be required to understand why the yield is lower than other techniques.

One of the proposed advantages of ISPR techniques is the increased product titers, reducing the downstream separation energy demand. Unfortunately, the product concentration in the organic phase is rarely stated; rather the total product quantity/concentration based on the fermenter volume is specified. Without this concentration, it is difficult to assess the extraction efficiency in the same manner as evaporative techniques, where the final condensate concentration is stated.

For an economically viable process, it is essential that the extractant is recyclable, meaning that the removal of ABE is straightforward. Unfortunately, removal of ABE from the extractant and regeneration of the extractant have not been discussed much in the literature. Roffler *et al*.^76^ developed a steam stripping or distillation system for the removal of ABE from oleyl alcohol as part of their economic assessment. This was successful as oleyl alcohol has a boiling point of 282–349°C, significantly higher than that of butanol. This technique has not been subjected to rigorous testing to understand the process and effects on the oleyl alcohol^76^. Vacuum distillation followed by flash separation has been suggested as an alternative method of separation, but this has not been proven experimentally.^85^ This is a very general investigation into the fermentation performance and energy requirements, not providing any indication whether flash separation of ABE from oleyl alcohol would be beneficial to the process. Lu and Li^86^ have suggested applying gas stripping to the extractant phase inside the fermenter. This regenerates the extractant while it is still in contact with the fermentation broth, removing the need for external distillation. The results of a bottle experiment have proven to be promising as the gas stripped extractant experiment had greater productivity and yield than a standard LLE experiment. The results are shown in Table 5. The ABE concentrations in the condensate from gas stripping stage ranged between 166 and 204 g ABE/L. This is a significant increase in product concentration compared to the butanol concentration in the extractant of 40 g butanol/L oleyl alcohol,^86^ although it is lower than the concentrations exhibited by the two‐stage gas process (500–700 g ABE/L^34^, ^36^). Lu and Li^86^ did not report any energy requirements for this system and Roffler *et al*.^70^ did not report the concentration of ABE after separation from the extractant, making a comparison with distillation or other ISPR techniques difficult.

To circumvent the re‐extraction step, Ishizaki *et al*.,^87^ Li *et al*.,^73^ and Yen and Wang^88^ have investigated the use of an extractant that would allow for direct use as a biofuel while in the extracted form. The use of biodiesel (methylated fatty acids, e.g., methylated crude palm oil) was shown to reduce the need for recovery from the extractant,^73^, ^87^, ^88^ as it produces an ABE‐enriched biodiesel, which significantly improves the quality of biodiesel with an increased cetane number and a reduced cold filter plugging point.^73^ The disadvantage with biodiesel is that it preferentially removes butyric acid from the fermentation, which is required by the bacteria to produce butanol.^73^ Yen and Wang^88^ suggest that the rate of butyric acid extraction is lower than the rate of assimilation by the bacteria; therefore, a negative impact was not seen, as confirmed by Table 5, the yield increased by 15% over the control fermentation without extraction by biodiesel. Ishizaki *et al*.^87^ demonstrated that the use of methylated crude palm oil is competitive in terms of fermentation characteristics compared to an extractive fermentation using oleyl alcohol, Table 5; with an 8% improvement in productivity and 5% yield improvements compared to 2% and 0% respective improvements with oleyl alcohol. The idea of using biodiesel as an extractant to create a superior biofuel is attractive and could form part of an ABE‐based biorefinery creating multiple products 89. If this were the case, the end use of the ABE produced needs to be considered when choosing an extractant.

### Perstraction (membrane extraction)

Perstraction is a development from liquid‐liquid extraction experiments. It works on the same principles of mass transfer of ABE from the aqueous phase to an organic solvent, but the organic solvent and fermentation broth are separated by a membrane. The ABE transfers across the membrane into the organic phase. It is very similar to pervaporation, but has a liquid on the permeate side to provide the “driving force” rather than a gas or vacuum. If the key criteria for LLE, outlined in Table 4, are not achieved, then LLE is not possible with the ABE fermentation. A membrane separating the two process streams can in principle overcome these problems.^90^

The main technique has been extraction into oleyl alcohol across a silicone membrane, as this has favorable partition characteristics for butanol.^25^, ^90^, ^91^, ^92^, ^93^ Qureshi and Maddox^92^ demonstrated in a batch fermentation that perstraction could easily improve the substrate utilization by 694%, productivity by 163%, and yield by 33%, Table 6. However Grobben *et al*.^93^ investigated the use of fatty acid methyl esters from sunflower oil, which would allow for the direct use of the extractant and biobutanol as a biofuel (this is similar to the work by Li *et al*.^73^ and Ishizaki *et al*.^87^). The use of fatty acid methyl esters did not match the performance by oleyl alcohol with a 40% decrease in productivity compared to the control fermentation and only a 16% increase in substrate utilization, Table 6. This could be due to the lower distribution coefficient for ABE in the fatty acid methyl esters (0.45, 1.1, and 0.05, respectively) compared to oleyl alcohol creating a lower driving force across the membrane. Jeon and Lee^90^ investigated two other solvents for the use of perstraction; polypropylene glycol and tributyrin. Table 6 shows that while the alternative solvents show improvement over the non‐integrated fermentation, productivity increases of 68% for polypropylene glycol and 42% for tributyrin, oleyl alcohol remains the best extractant for the recovery of ABE. When choosing possible extractants for perstraction, it seems that the same criteria for selecting an extractant for LLE were used in case of any back‐extraction of the solvent into the fermentation broth.^25^

**Table 6:** Free Cell ABE Fermentation Performance with *in situ* Product Recovery by Perstraction in an STR

Mode	Microorganism	Substrate (Concentration for ISPR)	% Substrate Increase for ISPR (vs. Control)	Productivity for ISPR (g ABE/Lh)	% Productivity Increase (vs. Control)	Yield for ISPR (g ABE/g Substrate)	% Yield Increase (vs. Control)	Membrane	Extractant	Ref.
Batch	*C. acetobutylicum* P262^a^	Lactose/Whey Permeate (227 g/L)	694%	0.21	163%	0.44	33%	Silicone tubing	Oleyl Alcohol	92
	*C. saccharoperbutyl‐acetonicum* N1‐4	Potato Glucose (89 g/L)	50%	0.32	−16%	0.21	−23%	PTFE	Oleyl Alcohol	95
	*C. saccharoperbutyl‐acetonicum* N1‐4	Potato Glucose (86 g/L)	45%	0.39	3%	0.23	−13%	PTFE	1‐Dodecanol	95
Fed‐Batch	*C. acetobutylicum* ATCC824	Corn Mash/Glucose (601 g/L)	902%	1.02	113%	0.36	23%	Silicone tubing	Oleyl Alcohol	90
	*C. acetobutylicum* ATCC824	Corn Mash/Glucose (422 g/L)	603%	0.81	69%	0.35	21%	Silicone tubing	Polypropylene glycol	90
	*C. acetobutylicum* ATCC824	Corn Mash/Glucose (155 g/L)	158%	0.68	42%	0.32	9%	Silicone tubing	Tributyrin	90
	*C. acetobutylicum* DSM1731 (ATCC8529)	Potato powder (92 g/L)	26%	1.00	59%	0.35	35%	Poly‐propylene	Oleyl Alcohol/Decanol	93
	*C. acetobutylicum* DSM1731 (ATCC8529)	Potato powder (85 g/L)	16%	0.38	−40%	0.32	23%	Polypropylene	Fatty acid methyl esters (MFA)	93
	*C. acetobutylicum* P262 a	Whey Permeate (123 g/L)	355%	0.24	243%	0.37	16%	Silicone tubing	Oleyl Alcohol	25
Contin‐uous	*C. acetobutylicum* ATCC824	Corn Mash/Glucose (2134 g/L) b	unknown	2.27	305%	0.33	2%	Silicone tubing	Oleyl Alcohol	91

a *C. acetobutylicum* P262 has since been reclassified as *C. saccharobutylicum* P262.^32^

b Dilution rate was 0.2 h^−1^.

There have been two examples, Shukla *et al*.^94^ and Tanaka *et al*.,^95^ where toxic solvents have been used in a perstraction system. Shukla *et al*.^94^ chose 2‐ethyl‐1‐hexanol as an extractant. 2‐ethyl‐1‐hexanol is known to be toxic to bacteria, but was considered less toxic than 1‐octanol, and therefore would be an acceptable extractant.^96^ The results of the toxicity tests of 1‐octanol or 2‐ethyl‐1‐hexanol were not published, so the degree of toxicity under the conditions described is unknown. Shukla *et al*.^94^ used a hollow fiber polypropylene membrane and did not report any ill effects from the use of this extractant; although an immobilized *C. acetobutylicum* was used for the fermentation, this could reduce the chances of the bacteria coming into contact with the solvent at the membrane interface. The only acknowledgement of using a toxic extractant was by Tanaka *et al*.,^95^ who chose 1‐dodecanol as an extractant. It was selected based on a high partition coefficient of 5.14, but it is unknown why 1‐dodecanol was chosen over other high‐distribution, toxic extractants such as 1‐octanol with a distribution coefficient of 5.6–7.33.^97^ The same levels of bacterial growth were seen when using 1‐dodecanol and oleyl alcohol as an extractant for perstraction. While the same levels of growth were seen and there was a slight increase in productivity (3%), there was a 13% decrease in yield compared to the control fermentation. The authors have not commented on this, as the maximum butanol productivity for both oleyl alcohol and 1‐dodecanol, 0.979 g/L.h was 1.25 times higher than the maximum butanol productivity for the control, 0.817 g/L.h.

One of the problems of LLE was the trade‐off between having a high partition coefficient and being non‐toxic to the bacteria. It was thought that the use of a membrane to aid the extractive process would allow for the use of extractants with higher partition coefficients. As seen in Table 6 from the description above most researchers have “played it safe” using extractants known to be non‐toxic to the bacteria. The earlier research appeared to indicate that some extractant is leaching across the membrane into the aqueous phase.^98^ Groot *et al*.^98^ believed that this was related to sorption of the solvent to the membrane. Some tests they performed using hexanol and silicone exhibited toxic effects to the fermentation, although data confirming this is not shown.^98^ Jeon and Lee^90^ stated that tributyrin had a partial inhibitory effect over time; therefore the fermentation with perstraction could not be run for as long, only 84 h consuming 154 g/L glucose compared to the oleyl alcohol based fermentation which operated for 209 h consuming 601 g/L glucose, resulting in the conclusion that non‐toxic solvents had to be used for perstraction. If this is true, one of the motivations for research into perstraction has been incorrect meaning there might be very little advantage of using perstraction rather than LLE. In contrast, Qureshi and Maddox^92^ suspected that back diffusion was a possible reason for the fermentation stopping, but later disregarded it, as it was shown that the fermentation stopped due to nutrient depletion. Combining this with the successful fermentations performed by Shukla *et al*.^94^ and Tanaka *et al*.^95^ using toxic extractants, there is no conclusive evidence that higher partition coefficient extractants cannot be used.

The majority of research has used silicone tubing as the membrane, Table 6,^25^, ^90^, ^91^, ^92^, ^98^, ^99^ because it is widely available and has acceptable mass transfer characteristics. It is also possible that it was chosen because it is readily available in the laboratory and easy to configure into an appropriate system.^25^, ^90^, ^91^, ^92^, ^98^ Jeon and Lee^90^ chose it as it has high permeability for butanol and acetone, can be autoclaved, has high mechanical strength, is easy to handle, biologically inert, compatible with many organic solvents, and had been used for pervaporation with the ABE fermentation.^90^ Qureshi and Maddox^92^ stated similar reasons for using a silicone membrane, including that silicone had been proven not to foul and there was no dead space for bacterial growth on the tubing. There has been very little comparison in terms of other membrane options. Only Groot *et al*.^98^ have compared possible membrane options, which were silicone, neoprene, and latex. Based on the mass transfer coefficient, silicone had the highest coefficient for all extractants tested compared to neoprene and latex. For hexanol, the corresponding mass transfer coefficient was 5.2 × 10^−7^ m/s for silicone, 0.4 × 10^−7^ m/s for neoprene, and 0.3 × 10^−7^ m/s for latex; meaning that the membrane choice will have a significant impact on the mass transfer in the system. No comparisons of polypropylene hollow fiber membranes and silicone have been performed. Grobben *et al*.^93^ have used an alternative polypropylene membrane. Polypropylene membranes appear comparable to silicone tubing (Table 6), but different bacterial strains and substrates have been used for the fermentation. Shukla *et al*.^94^ used a Celgard X20, a hydrophobic microporous hollow fiber membrane. It is suspected that this is also a polypropylene membrane which was commercially available at the time of research. Tanaka *et al*.^95^ chose a polytetrafluorothylene (PTFE) membrane as it is more hydrophobic than other membranes that have been used, therefore it should be more selective for ABE. It is evident that membranes for perstraction need to be optimized; as membrane development continues and become more commercially available, it is likely that more sophisticated industrially applicable membranes will become available.^92^

Comparing the fermentations in Table 6 with oleyl alcohol LLE fermentations in Table 5, not much difference can be seen in fermentation performance. Perstraction appears to enable higher fermentation productivities. Perstraction does show a greater, more consistent increase in yield, between the ISPR and control fermentation compared to the LLE fermentations; achieving an average yield of 0.33 g/g for integrated fermentations. This could be because the membrane reduces transfer of key nutrients and intermediates into the extractant phase. Similar to LLE, the recovery of ABE from the extractant is not considered, nor is the product concentration in the organic phase consistently reported. Qureshi and Maddox^92^ reported that the butanol concentration never exceeded 10 g butanol/L oleyl alcohol, although the extractant was replaced with fresh extractant five times during the fermentation. The extractant was replaced to limit the product build up in the fermenter, but this concentration is lower than that reported for LLE at 40 g butanol/L oleyl alcohol.^86^ Unless the extractant concentration can be increased or optimized for better fermentation performance, this lower extractant concentration is likely to increase the energy for distillation. In this scenario, the use of perstraction with non‐toxic extractants will have to be suitably justified to be applied to the ABE fermentation.

### Adsorption

Adsorption is the binding of a compound onto the surface of a solid adsorbent or resin. It is the oldest technique investigated for the use of ISPR from ABE fermentations. In 1948, Weizmann *et al*.^100^ first investigated butanol adsorption to relieve product inhibition and reduce the energy demand due to distillation.

A wide range of adsorbents have been used in conjunction with the butanol and the ABE fermentation, and this list is continually evolving as new, more complex adsorbents become available. Some of the adsorbents used are activated carbon,^100^, ^101^, ^102^, ^103^ silicalite or silicalite‐based zeolites,^23^, ^102^, ^104^, ^105^ and polymeric resins.^23^, ^101^, ^103^, ^106^, ^107^ The initial conclusion from Qureshi *et al*.^108^ was that silicalite adsorbents improved the fermentation the most as they have the ability to concentrate fermentation broth from 5 g butanol/L to 810 g butanol/L, but more recent work exhibits a tendency toward polymeric resins.^106^, ^109^, ^110^, ^111^ Over time, the adsorbents used have become increasingly complex with Cousin Saint Remi *et al*.^102^ recommending ZIF‐8, a metal organic framework adsorbent from Sigma‐Aldrich as a superior adsorbent to silicalite. The most recent work published on adsorption has moved back to the use of commercially available resins, along with selecting an activated carbon resin Norit ROW 0.8 to be combined with the fermentation broth rather than a polymeric resin such as Dowex Optipore L‐493 and SD‐2.^103^

The majority of the adsorbents have not been tested while coupled to an ABE fermentation, rather using a model ABE solution instead. Thus, there is a small amount of fermentation data to compare in Table 7. Yang *et al*.^112^ are one of the few who have investigated an adsorbent in conjunction with fermentation. They demonstrated that the addition of 30% resin to a fermentation can achieve 130% increase in productivity of the fermentation. When this was adapted to a fed‐batch fermentation with external column, the productivity increased by 233% for a single cycle adsorption, but 323% with multiple adsorption cycles. In the past two years, more research has focused on combining adsorption with the fermentation. Liu *et al*.^113^ combined the adsorption using KA‐I resin with an immobilized biofilm reactor. A membrane was used to ensure no biomass came into contact with the adsorbent. Two adsorption modes were investigated, selective for butanol and co‐adsorption of acetone, both these methods observed a reduction in productivity and yield by 37% and 9% for the butanol selective adsorption and 9% and 7% when acetone was co‐adsorbed. Lee *et al*.^114^ added the adsorbent directly to the fermenter. Fouling was not observed, but the authors commented on the potential physical interaction between the biomass and adsorbent being detrimental to the fermentation. The reasons suggested for this appear to be tenuous, but the mode of adsorption should be considered to minimize impact on the bacteria. This work demonstrated that in batch fermentations, using a modified *C. acetobutylicum* ATCC 824, the product concentration could be increased due to the adsorption of products, reducing toxicity. The final broth concentration reached 10 g butanol/L; this is the same as the fermentation with no ISPR, and the same yield was observed in both fermentations.^114^ Follow‐up work by Lee *et al*.^115^ using an *ex situ* adsorption column, combined with a fed‐batch fermentation using *C. beijerinckii* NCIMB 8052, saw no detrimental effects to the fermentation. The integrated and non‐integrated fermentation both had the same yield of 0.31, but the integrated fermentation had an increased total product concentration of 26 g ABE/L compared to 18.5 g ABE/L. No productivity was supplied for these fermentations.^115^ Wiehn *et al*.^116^ proposed an expanded bed adsorption process. This allows the fermentation broth to pass through the adsorbent without the need for microbial separation, due to the increased voidage space in the bed. A reduced biomass concentration was observed, compared to the control fermentation, but the overall fermentation metrics appeared positive with increases in both yield and productivity by 14% and 65%, respectively, Table 7. A limited degree of fouling was observed in the 72 h experiment, this could be a bigger issue in longer fermentations.^116^ Xue *et al*.^103^ used an activated carbon resin in both free cell STR fermentations and an immobilized bioreactor. The results of the free cell fermentation are shown in Table 7 with a productivity decrease of 3% and no change in the yield. The immobilized cell fermentation had a productivity increase of 22% and the same yield as the control fermentation. The batch fermentation only used a single cycle adsorbent, whereas the immobilized fermentation had three adsorbent cycles enabling greater product removal, hence a higher productivity. The immobilized fermentation eventually ceased due to a build‐up of acetone to 18 g/L in the fermentation broth, inhibiting the bacteria.^103^

**Table 7:** Free Cell ABE Fermentation Performance with *in situ* Product Recovery by Adsorption in an STR

Mode	Microorganism	Substrate (Concentration for ISPR)	% Substrate Increase for ISPR (vs. Control)	Productivity for ISPR (g/Lh)	% Productivity Increase (vs. Control)	Yield for ISPR (g/g)	% Yield Increase (vs. Control)	Adsorption Mode	Adsorbent	g adsorbent/mL liquor	Ref.
Batch	*C. acetobutylicum* ATCC824	Glucose (92 g/L)	22–47%	0.53	33%	0.32	3%	Batch (*in situ*)	Polyvinyl‐pyridine (PVP) resin	5%	112
				0.63	58%	0.32	2%			10%	
				0.74	85%	0.31	1%			20%	
				0.92	130%	0.32	3%			30%	
Fed‐Batch	*C. acetobutylicum* ATCC824	Glucose (190 g/L)	334%	1.33	233%	0.32	2%	Single cycle (*ex situ* column)	Polyvinyl‐pyridine (PVP) resin	n/a	117
	*C. acetobutylicum* ATCC824	Glucose (1199 g/L)	2636%	1.69	323%	0.32	4%	Cyclic (*ex situ* column)	Polyvinyl‐pyridine (PVP) resin	n/a	117
	*C. acetobutylicum* ATCC824	Glucose (180 g/L)	67%	0.72	14%	0.28	65%	Expanded Bed Adsorption (*ex situ)*	Poly (styrene‐co‐divinylbenzene) (Dowex Optipore L493)	n/a	116
	*C. acetobutylicum* JB200	Glucose (158 g/L)	267%	0.34 a	−3%	0.22 a	0%	Single cycle (*ex situ* column)	Activated Carbon (Norit ROW 0.8)	n/a	103

a Productivity of butanol production and butanol yield, rather than for total products.

Weizmann *et al*.,^100^ in agreement with Yang *et al*.^112^ and Lin *et al*.^109^ observed that the adsorption is competitive. Butanol is adsorbed in preference to acetone, for example. The order of preference for adsorption was ethanol as the weakest, followed by acetone, then butanol as the strongest.^109^ Yang *et al*.^112^ found that the order was ethanol, acetone, acetic acid, butanol, then butyric acid. This order is undesirable, as the butyric acid displaces the butanol, the desired product to be removed, and hinders the conversion of the butyric acid to butanol. Additionally, Xue *et al*.^103^ experienced the increased concentration of other products in the broth causing the fermentation to stop. This also raises questions as to whether any key nutrients are adsorbed during the process, which would be highly undesirable.

A downside of adsorption is that it is inherently a batch process, as the ABE has to bind to the adsorbent and then, once it has reached capacity, desorption has to occur. In many experiments, batch adsorption was performed,^101^, ^105^, ^107^, ^112^ meaning that once the adsorbent has reached capacity it can no longer relieve product inhibition. This indicates that the ratio of adsorbent to broth needs to be optimized, as the productivity varies with the quantity of adsorbent, Table 7. The alternative is operating a minimum of two external packed bed columns in a cyclic manner, allowing for one column to be adsorbing, while the other is desorbing.^23^, ^108^, ^110^, ^117^ Operation in this cyclic manner with a fed‐batch fermentation yielded a favorable fermentation productivity, Table 7.^103^, ^117^ This operating mode would reduce the adsorbent inventory required per fermentation, although the product removal would have to occur externally from the bioreactor. Ideally, the development of a continuous adsorption process (e.g., simulated moving bed adsorption) would be best suited, to allow the simplest removal of ABE and regeneration of adsorbent.

Once the adsorbent has reached its capacity the ABE needs to be removed and the adsorbent regenerated. The main methods of adsorption are through increasing the temperature,^102^, ^103^ displacement with steam^114^or another solvent, e.g., methanol,^109^, ^112^ or through vacuum evaporation.^106^, ^116^ The recovered butanol titers ranged from 43 to 167 g/L.^103^, ^114^ If the higher concentrations are consistently achievable, then the desorbed titers are similar to that achieved by LLE^86^, but still lower than the concentrations achieved in gas stripping.^36^

## Comparison of Techniques

Currently, no review has compared all possible ISPR techniques for ABE fermentations. One challenge is that it is difficult to make accurate comparisons between work performed by different groups, as different methods and procedures have been followed. This includes the use of different strains, media, reactor configurations, and mode of operation. To make a valid comparison between the different ISPR techniques, they have principally been compared in terms of their fermentation performance in STRs (see Tables 1−3 and 5−7). The downside of this approach is that not all techniques have been coupled to an ABE fermentation in an STR. Furthermore, there is significant variability in the data extracted within each ISPR technique.

From batch fermentations, it can be observed that every technique tested (gas stripping, vacuum fermentations, pervaporation, liquid–liquid extraction, perstraction, and adsorption) can have a positive effect on the fermentation. This is due to the removal of the butanol inhibition allowing for complete utilization of the substrate and the possibility of prolonged fermentations. This is seen by the significant increase in substrate utilization in Tables 1−3 and 5−7. Only in two batch fermentations was an increase in substrate not observed, Tables 3 and 5. In the case of pervaporation, no additional substrate was fed to the reactor and both the control and integrated fermentation consumed all the substrate supplied.^58^ For LLE, a decrease in substrate utilization was observed with a 50 wt% dodecanol in kerosene extractant; there was also a decrease in productivity and yield which could be related to toxicity of dodecanol to the bacteria.^74^, ^95^ The most prominent ISPR techniques for batch fermentations are gas stripping (see Table 1) and pervaporation (Table 3). It is difficult to compare different adsorption processes, as a major factor in the improvement in productivity is the quantity of adsorbent added to the broth which is not always reported, Table 7.

Where overcoming product inhibition has been successfully demonstrated for any technique, the next step is to perform a fed‐batch fermentation which increases substrate loading and fermentation time giving higher productivity. Increases of substrate between 100 and 900% compared to the control fermentation are common, Tables 1, 3, and 5−7. Fed batch data is only reported for: gas stripping, pervaporation, liquid–liquid extraction, perstraction, and adsorption. Adsorption, Table 7, enables the greatest improvement in productivity compared to standard batch fermentations, though the mechanism of adsorption contact has changed. This is closely followed by gas stripping, but the data in Table 1 has a degree of unreliability, as the productivity was calculated including estimated solvent losses.^5^ In all cases, a condenser was not sufficient to capture all the solvents produced, so the true productivity of the fermentation is unknown. This is a common problem with evaporative techniques due to the highly volatile nature of acetone and the high dilution of the vapors. Liquid–liquid extraction, however, appears to perform well with relatively repeatable increases in productivity and yield, Table 5.^75^

In literature, there have been some discrepancies between what is considered a fed‐batch and continuous process. There have been several occurrences where a fed‐batch fermentation has been called continuous in literature.^52^, ^58^ The continuous process described by Shin *et al*.^52^ and Li *et al*.^58^ is the same as the fed‐batch process described by Wu *et al*.^59^ and Qureshi and Blaschek.^68^ It must be recognized that for ISPR, typical process definitions no longer match the process. With a fed‐batch ISPR process, there is a feed in but there is also a product stream out. The product stream is not representative of the fermenter, in the same way it would be for a traditional continuous process, and the fermenter cannot be considered steady state as the concentrations (particularly biomass) change. Although often a concentrated feed is used to maintain a constant fermentation volume.

Continuous fermentations can be performed, but the continuous removal of fermentation broth leads to a lower increase in yield than for fed‐batch fermentations (Table 6). In spite of this reduced yield, greater consistency in improved productivity is seen. The reduction in yield for continuous fermentations is due to substrate removal via the outlet stream being accounted for as substrate consumption. This can have a significant effect on the process economics, as the substrate has the largest contribution to the cost of production.^1^ The dilution rate of a continuous fermentation controls the growth rate of the bacteria. From the limited data comparing continuous free cell fermentations, the dilution rate does not appear to affect the ISPR. Only gas stripping, pervaporation, and perstraction have been combined with continuous fermentation, with a greater focus on the application of fed‐batch fermentations. Interestingly, perstraction, Table 6, shows the greatest improvement in productivity for continuous fermentations closely followed by gas stripping, Table 1.

In recent years, there has been a growing trend to immobilized fermentations. A good example of this is Xue *et al*.^18^, ^33^, ^36^, ^103^ who have combined immobilized bioreactors with gas‐stripping and adsorption. Immobilized reactors have also been considered for combination with pervaporation and LLE.^80^, ^118^, ^119^ Immobilized reactors are not yet a realized commercial technology for the ABE fermentation,^39^ but in combination with an ISPR could allow for enhanced separation conditions, e.g., high temperatures,^19^ without being detrimental to the bacteria. Experimental comparisons of immobilized fermentations with ISPR need to be investigated to understand if immobilized fermentations should have an increased focus compared to free cell fermentations.

One of the factors driving the application of ISPR to the ABE fermentation is the potential energy reduction, due to increased ABE concentration going to downstream processing. The energy associated with separation is generally not considered alongside the experimental results. Xue *et al*.^120^ suggest that energy reductions will not be observed if the ISPR technique cannot concentrate the ABE to more than 40 wt%. This would allow removal of the beer stripper from product purification, which has the largest energy demand, concentrating the fermentation broth from 2 wt% ABE.^120^ None of the single‐single stage techniques have suggested they can concentrate the product to this concentration. The hybrid, two‐stage separation processes based upon an initial gas stripping stage are the only techniques to create an ABE solution greater than 40 wt%, with concentrations over 650 g ABE/L possible.^34^ To date, hybrid systems have only considered two‐stage gas stripping,^34^ combined gas‐stripping and pervaporation,^18^ and extractive gas stripping.^86^ There is the potential for many more hybrid or two‐stage separation systems to be designed. The economics of two‐stage systems will need to be considered and compared to a traditional batch and single‐stage ISPR process. The application of a single stage ISPR and beer stripper is effectively a two‐stage process; therefore the cost of implementations and operation could be a deciding factor for commercial implementation.

The review has compared six ISPR techniques based on available experimental data focusing on free cell fermentations. To mitigate the effects of various experimental methods, the % increase of substrate utilized, productivity, and yield was considered. The experimental data has successfully demonstrated that ISPR has a positive impact on the fermentation. The generation of models to represent the fermentation with integrated ISPR could help speed up developments in ISPR. They can help focus developments to the techniques which would provide the greatest improvements to the process. Experimental work can then be completed to validate the model results and confirm there are no biocompatibility issues. This will reduce the time and expense of testing every ISPR possibility experimentally, and provide a comparable baseline. The comparison of different experimental studies can also be improved through standardization of published experimental results for ISPR processes. This can be done by ensuring that enough data is provided to enable a mass balance of the process to be calculated, hence enabling an easier comparison; see supplementary material for a suggestive list of data to be included.

## Conclusion

From the comparison of STR fermentations, it is possible to say that all techniques exhibit improvements in fermentation productivity and that for different operating modes different techniques appear to be superior. For batch fermentations, gas stripping, and pervaporation were favorable, for fed‐batch: adsorption and gas stripping, and continuous perstraction has the greatest improvement. The use of novel two‐stage or hybrid techniques also needs to be considered, particularly their compatibility with free cell STR fermentations. This means that the decision on which technique to apply will be based on additional data such as energy consumption and an economic analysis. These should be considered alongside the fermentation data, and this would help to categorically state which is the best ISPR technique. Future work should include process optimization as part of trying new feedstocks, improved strains, or separating agents (e.g., membranes, adsorbents, and extractants).
